# Guided-acoustic stimulated Brillouin scattering in silicon nitride photonic circuits

**DOI:** 10.1126/sciadv.abq2196

**Published:** 2022-10-07

**Authors:** Roel Botter, Kaixuan Ye, Yvan Klaver, Radius Suryadharma, Okky Daulay, Gaojian Liu, Jasper van den Hoogen, Lou Kanger, Peter van der Slot, Edwin Klein, Marcel Hoekman, Chris Roeloffzen, Yang Liu, David Marpaung

**Affiliations:** ^1^ Nonlinear Nanophotonics, MESA+ Institute of Nanotechnology, University of Twente, Enschede, Netherlands.; ^2^ LioniX International, Enschede, Netherlands.; ^3^ Institute of Physics, Swiss Federal Institute of Technology Lausanne (EPFL), CH-1015 Lausanne, Switzerland.

## Abstract

Coherent optomechanical interaction known as stimulated Brillouin scattering (SBS) can enable ultrahigh resolution signal processing and narrow-linewidth lasers. SBS has recently been studied extensively in integrated waveguides; however, many implementations rely on complicated fabrication schemes. The absence of SBS in standard and mature fabrication platforms prevents its large-scale circuit integration. Notably, SBS in the emerging silicon nitride (Si_3_N_4_) photonic integration platform is currently out of reach because of the lack of acoustic guidance. Here, we demonstrate advanced control of backward SBS in multilayer Si_3_N_4_ waveguides. By optimizing the separation between two Si_3_N_4_ layers, we unlock acoustic waveguiding in this platform, potentially leading up to 15× higher Brillouin gain coefficient than previously possible in Si_3_N_4_ waveguides. We use the enhanced SBS gain to demonstrate a high-rejection microwave photonic notch filter. This demonstration opens a path to achieving Brillouin-based photonic circuits in a standard, low-loss Si_3_N_4_ platform.

## Teaser

Double-stripe waveguide opens the way to harness stimulated Brillouin scattering in a standard, low-loss Si_3_N_4_ platform.

## INTRODUCTION

Coherent control of light through optomechanical interactions (*1*–*3*) with acoustic waves and vibrations is a burgeoning field with a wide range of applications, from quantum optics to telecommunications. In particular, stimulated Brillouin scattering (SBS) (*4*, *5*), an optomechanical interaction between light and gigahertz sound waves, is currently revolutionizing integrated optics (*3*). SBS spectrally manifests in narrowband (tens of megahertz) gain resonance, shifted in frequency by about 10 GHz from the pump frequency, making it a unique filter and amplifier with technological importance in next-generation optical and radio communications (*6*–*10*), high-precision sensors (*11*–*14*), low-threshold narrow-linewidth lasers (*15*–*18*), and nonreciprocal light propagation (*19*–*22*).

Recently, there are notable interests in inducing and inhibiting SBS in centimeter-length chip-scale photonic devices (*3*, *21*, *23*–*28*), with experimental demonstrations in various integrated photonic platforms, including chalcogenide glasses (*23*), silicon (*26*), doped-silica (*29*), gallium arsenide (*30*), and aluminum nitride (*31*). Despite encouraging results, however, SBS devices are still singular and difficult to integrate into large-scale and versatile circuits because of the need for unconventional materials (*23*) or suspended structures (*26*).

Harnessing SBS in silicon nitride, which is an emerging low-loss (*32*–*35*) and versatile (*36*–*38*) integration platform, can unlock promising technologies including lasers (*15*, *39*–*41*), frequency combs (*42*–*45*), microwave photonics (*6*, *38*, *46*), and on-chip amplifiers (*47*). However, the investigation of SBS in standard silicon nitride waveguides is still in its infancy, and the measured effect is relatively weak. Two recent reports of SBS in thin (40 nm) (*15*) and thick (800 nm) (*48*) silicon nitride waveguides reveal gain coefficients of 0.1 and 0.07 m^−1^ W^−1^, respectively. Both demonstrations are plagued by acoustic leakage from the silicon nitride core to the surrounding silicon oxide cladding, preventing the SBS enhancement in nanophotonic waveguides (*3*) (see the Supplementary Materials for the comparison between different SBS platforms).

In this work, we demonstrate guided-acoustic SBS in multilayer silicon nitride nanophotonic circuits. We use multilayer silicon nitride waveguides to confine both the optical and the gigahertz acoustic waves, solving the problem of acoustic leakage faced by waveguides with a single silicon nitride core. We show the feasibility of tailoring the cross section of the multilayer silicon nitride waveguides for on-demand enhancement or inhibition of SBS, making it possible for circuit-level selective SBS generation. We further demonstrate an application in radio frequency (RF) signal processing through a notch filter with a high rejection of 66 dB using only 0.4 dB of SBS gain. This constitutes a substantial advancement of SBS amplification and filtering in a single-pass silicon nitride waveguide that is previously unreachable because of the insufficient Brillouin gain. Our results are realized in a standard, low-loss silicon nitride platform, opening the way to integrating SBS elements in a large-scale circuit and intersecting it with emerging technologies including tunable lasers, frequency combs, and programmable photonic circuits.

## RESULTS

### Enhancement and inhibition of SBS

**Fig. 1.**
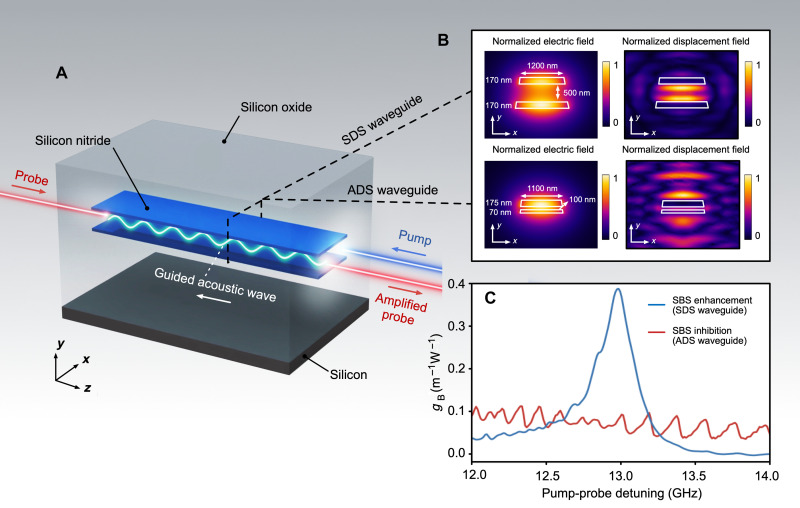
Guided-acoustic Brillouin scattering in multilayer silicon nitride waveguides. (**A**) Artistic representation of the multilayer silicon nitride waveguide, showing the enhanced backward SBS process with the acoustic wave guided between the silicon nitride layers. (**B**) Simulated optical modes and acoustic responses of the SDS and ADS waveguides, respectively. SDS and ADS are two variances of the multilayer silicon nitride waveguides. (**C**) The calculated Brillouin gain coefficients of the standard SDS and ADS waveguides. The SDS waveguide shows enhanced SBS with a gain coefficient three to five times larger than previously demonstrated in silicon nitride, while the ADS waveguide shows inhibited SBS, with a Brillouin gain coefficient below 0.1 m^−1^ W^−1^.

The enhancement of backward SBS in our multilayer silicon nitride waveguides is illustrated in Fig. 1A. Scattering of the pump from the acoustic wave, which is guided between the silicon nitride layers, results in amplification of the Stokes probe wave. Precise control of the separation between the silicon nitride layers tailors the acoustic waveguiding, hence enabling on-demand enhancement and inhibition of SBS in these waveguides.

Here, we investigate two waveguide geometries with different separations between the silicon nitride layers. The symmetric double stripe (SDS) waveguide has a separation of 500 nm, while the asymmetric double stripe (ADS) waveguide has a separation of only 100 nm (See Methods for details of the waveguide structures). Both geometries are standard waveguide structures that have been optimized for low-loss and high-density photonic circuits (*37*), which allows for the integration of SBS with a library of photonic devices with complementary functionalities.

We carry out finite element simulations of the optical and acoustic modes, along with the calculated Brillouin gain coefficient (see Supplementary Materials for details of modeling), to reveal distinct SBS optoacoustic interactions in SDS and ADS structures. The results are depicted in Fig. 1B. The SDS shows waveguiding of fundamental transverse electric optical mode at 1550 nm along with SBS phase–matched 12.99-GHz acoustic wave guided between the silicon nitride layers, leading to enhanced optoacoustic overlap. On the other hand, the ADS waveguide shows the absence of such acoustic waveguiding, and the optoacoustic overlap is markedly reduced.

The SBS gain is strongly affected by acoustic waveguiding (*5*, *49*), and this is evident in the calculated gain coefficient, *g*_B_, for the SDS and ADS structures, as shown in Fig. 1C. The SDS waveguide shows a clear peak of *g*_B_ = 0.38 m^−1^ W^−1^ at 12.99 GHz. This calculated value is three to five times larger than previously reported in thin (*15*) and thick (*48*) silicon nitride waveguides, indicating the potential of enhancing SBS in multilayer waveguides. In contrast, the ADS waveguide shows no clear SBS peak, with the calculated *g*_B_ staying below 0.1 m^−1^ W^−1^ over a broad frequency range of 12 to 14 GHz. Inhibition of SBS in this structure is due to the thin separation of the silicon nitride layers that prevents acoustic waveguiding, similar to previous observations in thin (*15*) and thick (*48*) silicon nitride waveguides. This stark difference in SBS response occurs in two structures with similar effective indices, bend radii, and propagation losses.

### Experimental results

**Fig. 2.**
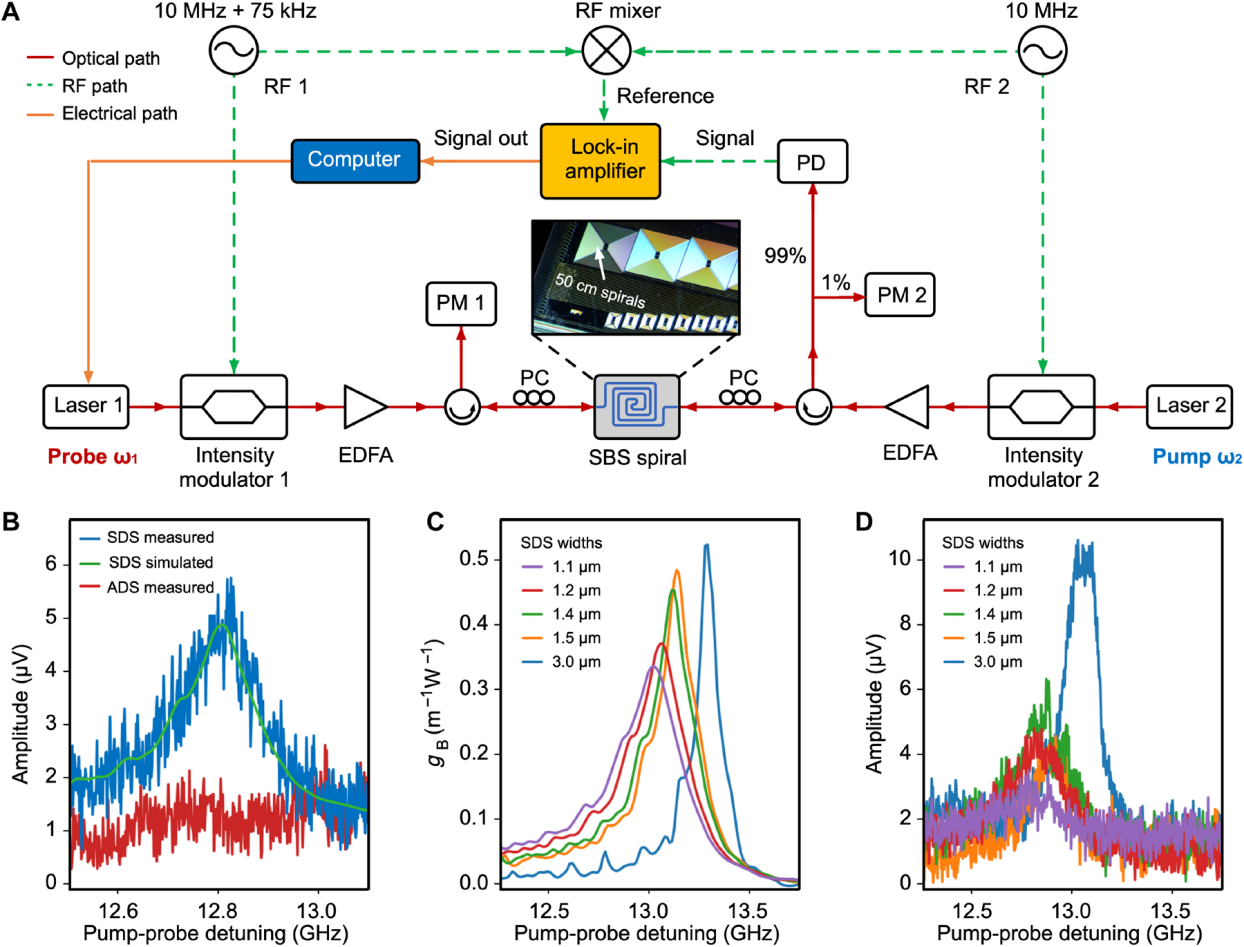
Characterization of SBS in multilayer silicon nitride waveguides. (**A**) Schematic of the setup used for the SBS characterization. EDFA, erbium-doped fiber amplifier; PM, power meter; PC, polarization controller; PD, photodetector; RF, radiofrequency signal generator. See Methods for details of the experiment. (**B**) Measured SBS gain spectra of the standard SDS and ADS waveguides. The SDS simulation result has been overlayed on the measurement response. (**C**) Simulation results of the SBS gain spectra in SDS waveguides for the varying waveguide widths represented in the available samples. The highest gain coefficient of 0.53 m^−1^ W^−1^ was predicted for the 3-μm-wide waveguide. (**D**) Measured SBS gain spectra for SDS waveguides with varying waveguide widths, showing very good agreement with the trend predicted from simulations. The waveguide loss ranges from 0.19 to 0.23 dB/cm. The highest SBS gain is around 0.40 m^−1^ W^−1^ from the 3-μm-wide waveguide.

We devise an experimental apparatus diagrammed in Fig. 2A to measure SBS in our samples. The samples are 50-cm-long spirals of both SDS and ADS waveguides, with losses of 0.22 and 0.15 dB/cm, respectively, as shown in the inset. For high sensitivity, we use the dual intensity modulation pump-probe technique, which was previously used in Brillouin spectroscopy (*50*) and later on adapted for the detection of SBS in thick silicon nitride waveguides (*48*) (see the Supplementary Materials for details of the experiments).

The measured SBS gain responses of both the standard SDS and ADS waveguides are shown in Fig. 2B. The response from the SDS waveguide matches well with our simulation results. The measured Brillouin gain coefficient is 0.24 m^−1^ W^−1^, 2.5× higher compared to previously reported values in silicon nitride waveguides (*15*, *48*) (see Methods for details of the gain calculation). On the other hand, the measured response from the ADS waveguide is too weak to be detected, which is in agreement with the SBS inhibition predicted from the simulations.

Increasing the width of the SDS waveguides leads to improvement of the acoustic waveguiding and enhancement of the SBS gain. As depicted in the simulation results in Fig. 2C, a Brillouin gain coefficient can be increased up to 0.53 m^−1^ W^−1^ with a 3.0-μm-wide SDS waveguide. Figure 2D shows the measured SBS responses of the SDS waveguides with different widths, confirming the trend predicted from the simulations. We measured a gain coefficient of 0.40 m^−1^ W^−1^ from a 3.0-μm-wide SDS waveguide, with a linewidth of 130 MHz. The Supplementary Materials summarizes the comparison between the simulation and experimental results.

### RF photonic notch filter demonstration

**Fig. 3.**
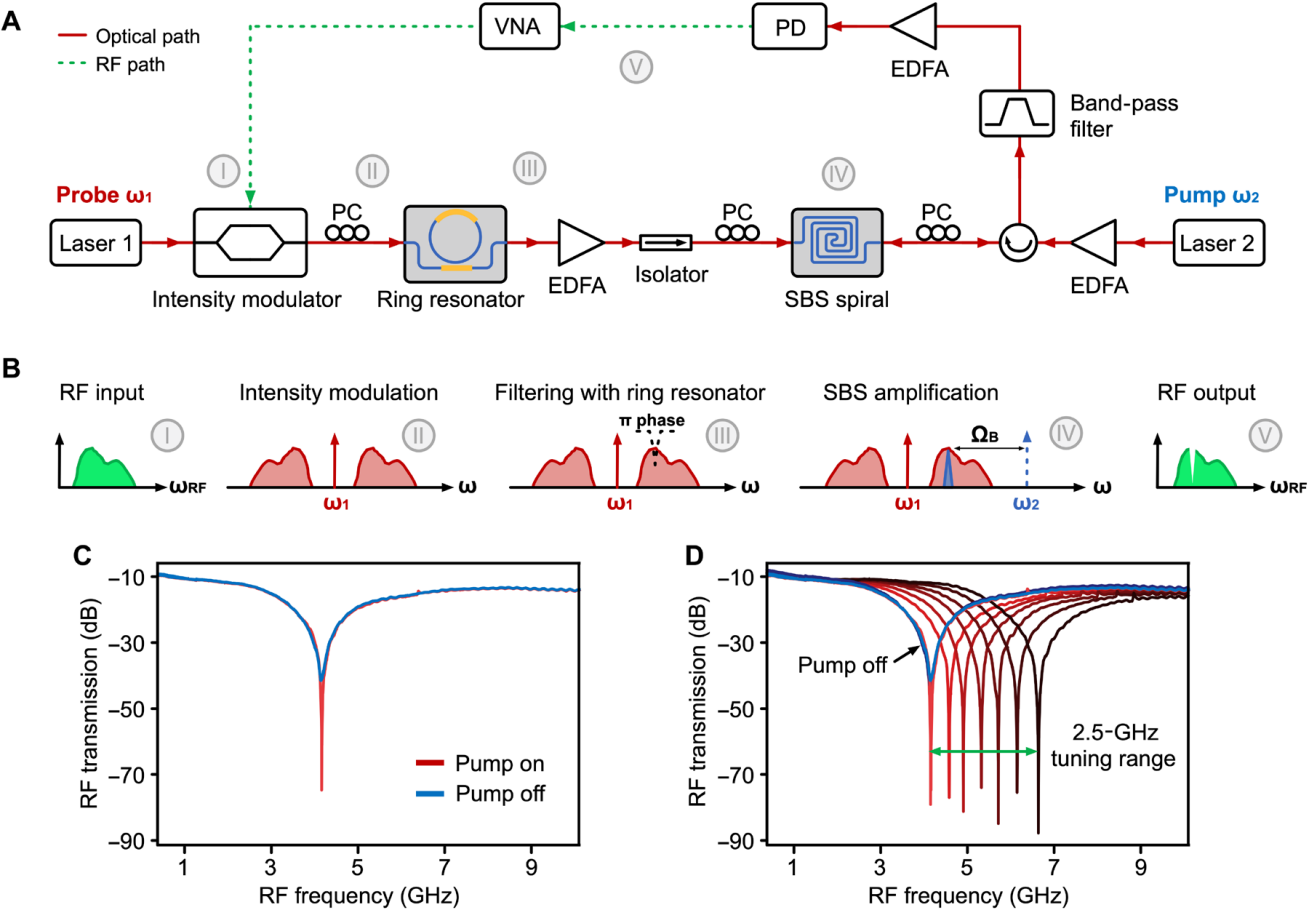
Microwave photonic notch filter using SBS in a silicon nitride waveguide. (**A**) Schematic of the experimental setup to demonstrate the microwave photonics notch filter. VNA, vector network analyzer. See Supplementary Note B for details of the experiment. (**B**) RF and optical spectra at different points of the signal path: (I) input RF signal. (II) Intensity-modulated signal. (III) The signal after passing through the over-coupled ring resonator. (IV) The SBS gain is applied to the signal. (V) The resulting RF output signal. (**C**) The measured high-rejection RF photonic notch filter responses. (**D**) Central frequency tuning of the notch filter.

One of the key applications of chip-based SBS is microwave photonic signal processing (*6*, *9*, *51*). To investigate the potential applications in microwave photonics with the SBS gain from the SDS waveguides, we demonstrate an RF cancellation notch filter (*7*, *52*, *53*) with the setup diagrammed in Fig. 3A. Here, we combine optical filtering response from an SDS ring resonator with SBS gain from a 3.0-μm-wide SDS waveguide to achieve enhanced filter rejection from minute SBS gain (*54*, *55*).

Figure 3B illustrates the working principle of the RF photonic notch filter. The RF input (I) is intensity-modulated onto the probe laser with two sidebands of equal phase and amplitude (II). An overcoupled SDS ring resonator is used to filter the upper sideband and, at the same time, induce a π phase shift at the frequency shifted from the pump by the Brillouin frequency shift Ω_B_ (III). After amplification with an erbium-doped fiber amplifier (EDFA), the signal is injected into an SBS spiral. The SBS gain of the waveguide compensates for the notch response from the ring, making sidebands equal in amplitude but with a π phase shift only at the Brillouin shift frequency (IV). The processed signal is then sent to a photodiode, resulting in a notch in the RF response due to the destructive interference (V).

The measured RF photonic notch filter response is shown in Fig. 3C. High notch rejection of 66 dB and a 3-dB bandwidth of 2.4 GHz are obtained from a mere 0.4 dB of on-chip SBS gain. The bandwidth of this filter is currently limited by the synthesized ring response, which is tuned to an overcoupled response with a shallow rejection (*55*). We expect that narrower bandwidth can be achieved with increased SBS gain. Figure 3D shows that the central frequency of the notch filter can also be tuned over 2.5-GHz range. Further, we measured a noise figure of 43.7 dB and a dynamic range of 100.5 dB/Hz^2/3^ of this filter (see the Supplementary Materials for details of the filter performances), which is already comparable with the SBS notch filter in silicon waveguide (*56*). This result constitutes the first signal processing demonstration of SBS in silicon nitride waveguides and points toward the potential of unlocking unique Brillouin signal processing capabilities in a mature silicon nitride platform.

## DISCUSSION

We observe the first signature of SBS in multilayer silicon nitride waveguides. Enhancement or inhibition of SBS depends on the cross section, more specifically, the separation between the silicon nitride layers. The SDS geometry has a separation of 500 nm, showing enhanced SBS with the acoustic waves guided between the silicon nitride layers. In contrast, the ADS has a smaller separation, which prevents acoustic waveguiding and inhibits SBS. Moreover, we show through simulations and experiments that tailoring the width of the SDS waveguide can improve acoustic waveguiding and lead to enhanced SBS.

We show that the measured SBS response in our silicon nitride waveguides can readily be used for RF photonic signal processing and filtering. Using a mere 0.4 dB of SBS peak gain from the SDS waveguide, we demonstrated an RF photonic notch filter with a rejection of 66 dB and a 3-dB bandwidth of 2.4 GHz.

Further optimization of the multilayer silicon nitride waveguide can lead to substantial SBS enhancement (see the Supplementary Materials for details of the optimization). By only tuning the geometry parameters, we can achieve a Brillouin gain coefficient of 1.2 m^−1^ W^−1^ with a Brillouin frequency shift of 14 GHz and a linewidth of 35 MHz, representing 15× gain enhancement compared to previously reported values (*48*). Moreover, adding sidewalls to the double-stripe waveguide to form a box-shaped waveguide cross section can contribute to better acoustic confinement and a Brillouin gain coefficient of 1.4 m^−1^ W^−1^ can be realized (see the Supplementary Materials for simulation results of the box-shaped waveguide).

**Fig. 4.**
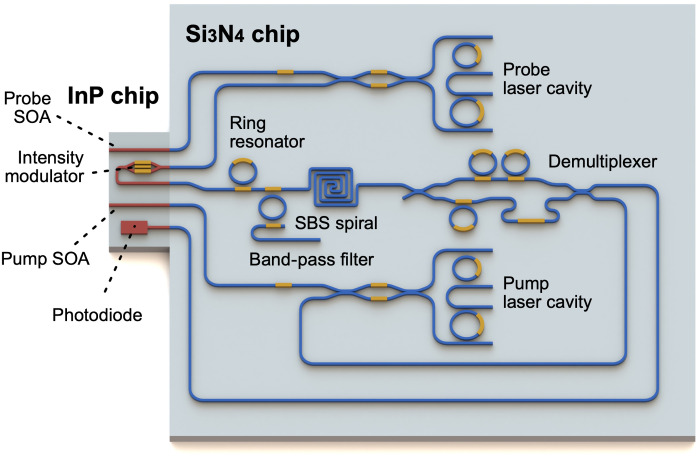
Artistic impression of an all-integrated SBS microwave photonic filter. InP, indium phosphide; Si_3_N_4_, silicon nitride; SOA, semiconductor optical amplifier.

The optimized waveguide can be the basis of unique SBS devices embedded in large-scale and low-loss silicon nitride circuits. As shown in Fig. 4, the optimized SBS waveguides can be integrated with advanced microwave photonic spectral shaping circuits (*57*, *58*) to form an all-integrated high-resolution RF filter with a low insertion loss, low noise figure, and ultrahigh dynamic range. Furthermore, the development of high-power erbium-doped waveguide amplifiers in silicon nitride photonic integrated circuits (*47*) will overcome the current necessities of off-chip EDFAs and, at the same time, enable increased link gain. Last, we expect that the implementation of SBS lasers in this optimized silicon nitride waveguide geometry can open the path to full integration of the pump and the SBS lasers (see the Supplementary Materials for SBS laser threshold calculation) and can intersect SBS lasers with Kerr microcombs (*59*–*62*) for versatile sources in silicon nitride platform.

## METHODS

### Silicon nitride waveguide fabrication

Our waveguides are fabricated using the standard LioniX TriPleX process (*35*, *37*). First, an 8-μm SiO_2_ layer is grown through wet thermal oxidation of the single-crystal silicon substrate at 1000°C. Then, low-pressure chemical vapor deposition (LPCVD) is used for the Si_3_N_4_ layers, as well as the intermediate SiO_2_ layer. For the SDS waveguides, both Si3N4 layers are 170 nm thick. The intermediate SiO_2_ layer is 500 nm thick. In the case of the ADS waveguides, the bottom Si_3_N_4_ layer is 75 nm thick, and the top layer is 175 nm thick. These layers are separated by 100 nm of SiO_2_.

After the layers have been deposited, the waveguides are patterned using contact lithography and processed with reactive ion etching. Then, the waveguides are covered with an additional 1.5-μm-thick layer of SiO_2_ through LPCVD. The full 8-μm thickness of the cladding is then achieved using plasma-enhanced chemical vapor deposition (PECVD).

### Determining the Brillouin gain coefficient

The SBS gain in a waveguide is determined by

$$G=e^{g_{B}L_{\text{eff}}P_{\text{pump}}}$$ (1)

where *g*_B_ is the Brillouin gain coefficient in m^−1^ W^−1^, *L*_eff_ is the effective length, and *P*_pump_ is the pump power in the waveguide.

The effective length of a waveguide is calculated using

$$L_{\text{eff}}=\frac{1-e^{-\alpha L}}{\alpha }$$ (2)

where α is the propagation loss and *L* is the actual waveguide length.

By using Eq. 1, and taking the small signal approximation, we can calculate the gain coefficient using

$$g_{B,\text{SDS}}=\frac{V_{\text{SDS}}}{V_{\text{fiber}}}\frac{g_{B,\text{fiber}}L_{\text{eff},\text{fiber}}P_{\text{pump},\text{fiber}}}{L_{\text{eff},\text{SDS}}P_{\text{pump},\text{SDS}}}$$ (3)

Here, *V* denotes the signal voltage measured by the lock-in amplifier, and the subscripts fiber and SDS refer to the properties of the fiber and chip used in this experiment.

## Supplementary Materials

### This PDF file includes:

Supplementary Text Figs. S1 to S11 Tables S1 to S8 References
