# Supplementary material for: Guided-acoustic stimulated Brillouin scattering in silicon nitride photonic circuits
Source: Sci Adv. 2022 Oct 7;8(40):eabq2196. doi: 10.1126/sciadv.abq2196 (PMC9544327; doi:10.1126/sciadv.abq2196)
Supplement: Supplementary file 1 — Supplementary Text Figs. S1 to S11 Tables S1 to S8 References [file sciadv.abq2196_sm.pdf]

Science Advances
AAAS

Supplementary Materials for

Guided-acoustic stimulated Brillouin scattering in silicon nitride photonic circuits

Roel Botter *et al.*

Corresponding author: David Marpaung, david.marpaung@utwente.nl

Sci. Adv. **8**, eabq2196 (2022)
DOI: 10.1126/sciadv.abq2196

This PDF file includes:

Supplementary Text
Figs. S1 to S11
Tables S1 to S8
References

SUPPLEMENTARY NOTE A: SIMULATION AND OPTIMIZATION

This supplementary note describes our simulation model. Based on our model, we discuss the influence of each waveguide parameter on the Brillouin gain coefficient. We also discuss the optimization procedures with a genetic algorithm. We verify our model by comparing the results to those of earlier published work.

Simulation Setup and Method

Fig. S1a shows the cross section of our optical simulation model, which matches the real SDS waveguide. In the 2D acoustic simulation model shown in Fig. S1b, everything is the same except that the additional perfectly matched layers (PML) to significantly reduce reflections from the borders. We apply COMSOL Multiphysics for both optical and acoustic simulations, and material properties applied in our model are listed in Table S1. The procedure of our simulations is as follows. First, we simulate the optical modes of the pump and probe. Then, we calculate the electrostrictive stress tensor and optical forces induced by the electrostriction effect. The influence of radiation pressure is ignored here because the refractive index between silicon nitride and silicon oxide is small. After the optical simulation, we map the electrostrictive stress tensor and optical forces to the acoustic model. Finally, the Brillouin gain coefficient is calculated based on the overlap between optical forces ($\tilde{\mathbf{f}}_\mathrm{n}$) and acoustic responses ($\dot{\tilde{\mathbf{u}}}_\mathrm{n}$) [5]:

$$g_B(\Omega) = 2 \cdot \left(\frac{\omega_s}{\Omega}\right) \int_{\mathrm{wg}} \mathrm{Re}\left[\tilde{\mathbf{f}}_\mathrm{n}(\mathbf{x},\mathbf{y}) \cdot \dot{\tilde{\mathbf{u}}}^*_\mathrm{n}(\mathbf{x},\mathbf{y})\right] dA, \tag{S1}$$

where ω_s is angular frequency of the Stokes wave and Ω is the angular frequency of the excited acoustic mode.

The peak value of the Brillouin gain coefficient can also be simplified to [63]:

$$g_\mathrm{B} = \frac{4\pi^2\gamma_\mathrm{e}^2\eta}{n_\mathrm{p} c \lambda_p^2 \rho_0 v_\mathrm{A} \Gamma_\mathrm{B} A_\mathrm{eff}} \tag{S2}$$

where γ_e is the electrostrictive constant, η the opto-acoustic overlap, n_p the refractive index, λ_p the pump wavelength, ρ_0 the density, v_a the speed of sound, Γ_B the Brillouin linewidth, and A_eff the effective area.

We also apply the 3D acoustic simulation model shown in Fig. S1c to investigate the propagation of the excited acoustic mode. The acoustic simulation cross section is extruded for 10 wavelengths and followed by PML in the z direction. We map the acoustic response from the 2D model as initial condition and monitor the wave as it propagates along the z-direction. As depicted in Fig. S2, the acoustic field stays between the silicon nitride layers beyond more than 5 µm of propagation (10 × the acoustic wavelength), clearly showing acoustic waveguiding.

Influences of different parameters on SBS gain

The Brillouin gain coefficient is related to multiple waveguide parameters. To investigate the influences of different parameters, we first find the optimized geometry using a genetic algorithm, as described in the next section, then sweep each single parameter to get the corresponding SBS gain profiles. The optimized geometry parmeters are t_int = 420 nm, t_g = 200 nm, t_c = 7350 nm, and w = 3100 nm. Fig. S3a–d shows the variation of g_B when we sweep waveguide width w, stripe thickness t_g, stripe separation t_int and cladding thickness t_c separately. As w increases, the SBS gain first increases because of improved acoustic confinement, then it saturates when the increased effective mode areas of both the optical and acoustic field cancel out the benefit from improved acoustic confinement. As t_g increases, the SBS gain also increases at first, due to better overlap between optical and acoustic fields. However, the optical mode will be more concentrated within the stripes as t_g increases further, leading to a reduced overlap and a smaller SBS gain. The oscillatory behavior of the gain coefficient with t_int and t_c is believed to be a result of a change of the resonance condition of the acoustic mode in the intermediate layer.

TABLE S1. Material properties applied in our simulation model.

	**Refractive Index**	**Poisson Ratio**	**Young's Modulus** (GPa)	**Density** (kg/m^3)	**p$_{12}$**
Si_3N_4 [48]	1.98	0.23	201	3020	0.047
SiO_2 [64]	1.45	0.19	74	2240	0.27
Si [64]	3.48	0.275	180	2330	0.017

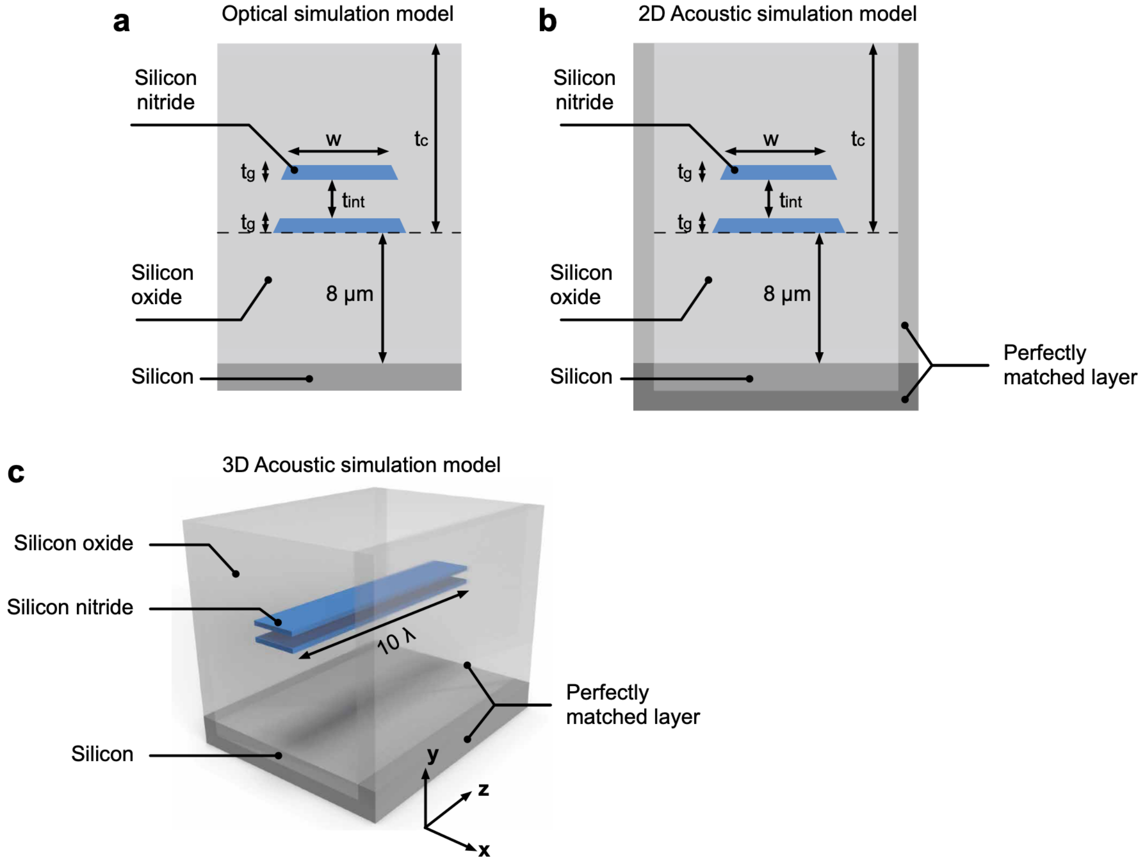

Fig. S1. Setup of our simulation model. **a**, Optical simulation cross section; **b**, Acoustic simulation cross section; **c**, 3D acoustic simulation model.

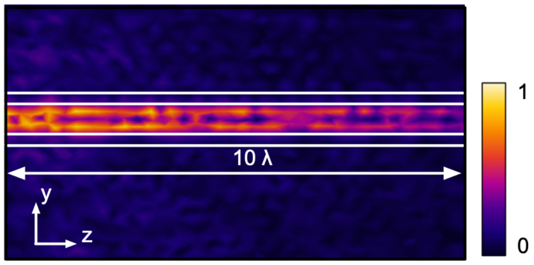

Fig. S2. Simulated acoustic field strength as a function of a propagation distance. The geometry used is the standard SDS waveguide, as shown in Fig. 1 of the main text.

Optimization with Genetic Algorithm

We apply a genetic algorithm to optimize the geometry of the waveguide for a higher Brillouin gain coefficient [65]. In our gene pool, t_g ranges from 100 nm to 300 nm with a step of 10 nm, t_{int} ranges from 200 nm to 700 nm with a step of 10 nm, w ranges from 500 nm to 5000 nm with a step of 100 nm, and t_c ranges from 7 μm to 9 μm with a step of 20 nm. These ranges and step sizes result in nearly 5 million combinations, which requires an unreasonably long time to process. Instead, we simulate 1760 SDS geometries based on a 14-round evolution genetic algorithm. The genetic algorithm is carried out as follow. First, we randomly generate 200 candidates from the gene pool. We simulate the SBS gain of each candidate from 12 GHz to 14 GHz. Then, We select 80 elites with the highest peak g_B. After that, we generate 80 kids by randomly pairing 80 elites. For each pair gives birth to two kids. One of them inherits three genes from the mother and one gene from the father, while the other kids inherits the opposite. During

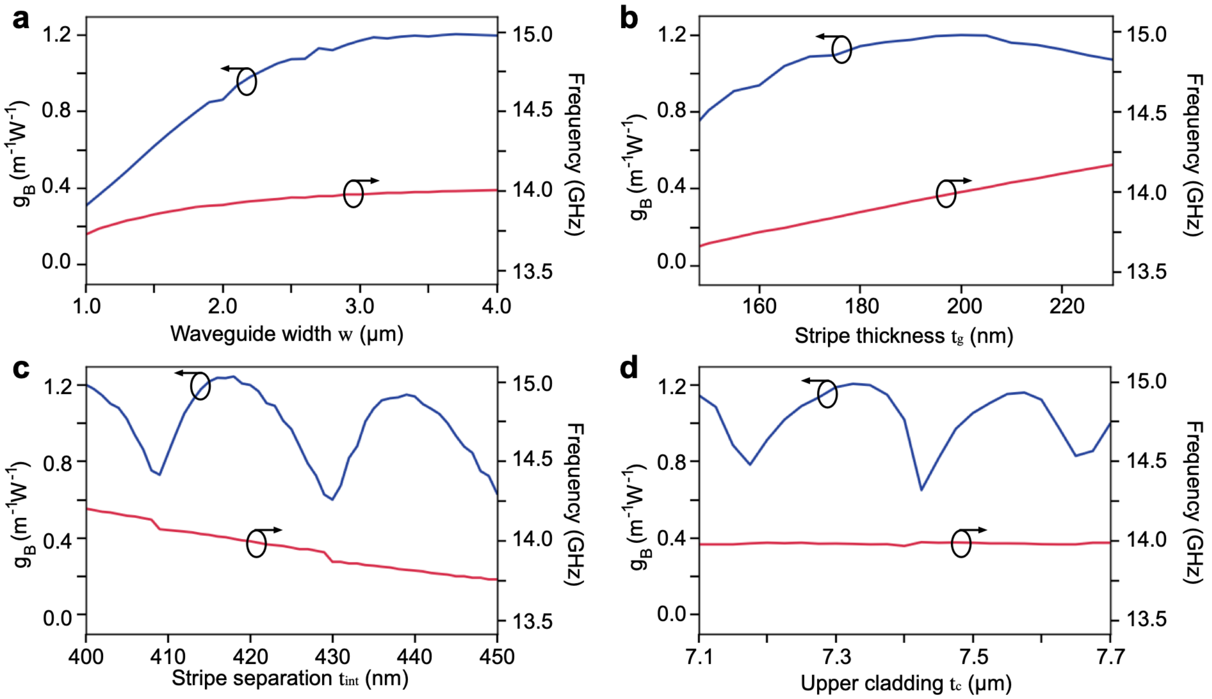

Fig. S3. **The Brillouin gain profile as a function of different parameters.** **a**, separation between the two stripes; **b**, stripe thickness; **c**, stripe width; **d**, cladding thickness.

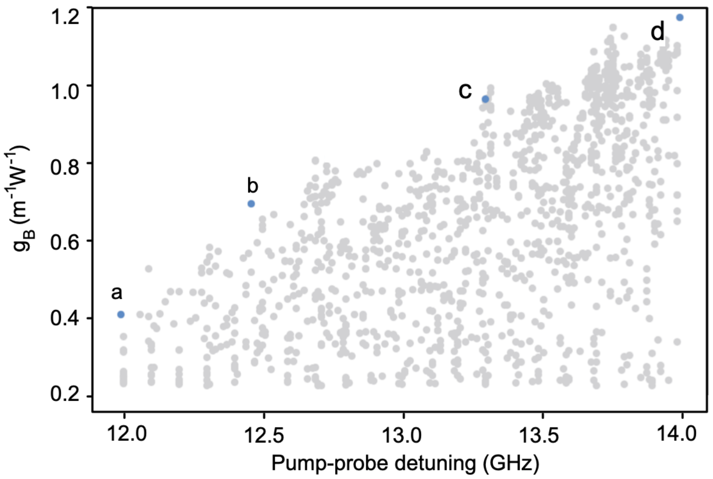

Fig. S4. **The SBS gain and Brillouin shift frequencies of all geometries simulated with the genetic algorithm.** The geometries selected in Fig. S5 are indicated by blue dots labeled a–d.

the inheritance, there is a 15% chance that one of the genes mutates, i.e., the value of that gene increases or decreases by 20%. Finally, we also introduce 40 new candidates from the gene pool to increase the variety. Both parents and kids and the new candidates will then enter the next round of evolution.

Fig. S4 shows the peak Brillouin gain coefficients versus the corresponding Brillouin shift of all the structures simulated with the genetic algorithm. The highest gain coefficient is around 1.2 $m^{-1}W^{-1}$ at 14 GHz. Fig. S5 shows the normalized acoustic displacement and electric fields, as well as gain profiles of some selected geometries that are labelled a–d in Fig. S4. We observe the linewidth of the SBS gain spectrum narrows down as the gain coefficient increases, which indicates a longer acoustic lifetime. The extended acoustic lifetime would primarily be achieved by reduced radiated acoustics, signifying better acoustic confinement in the intermediate layer.

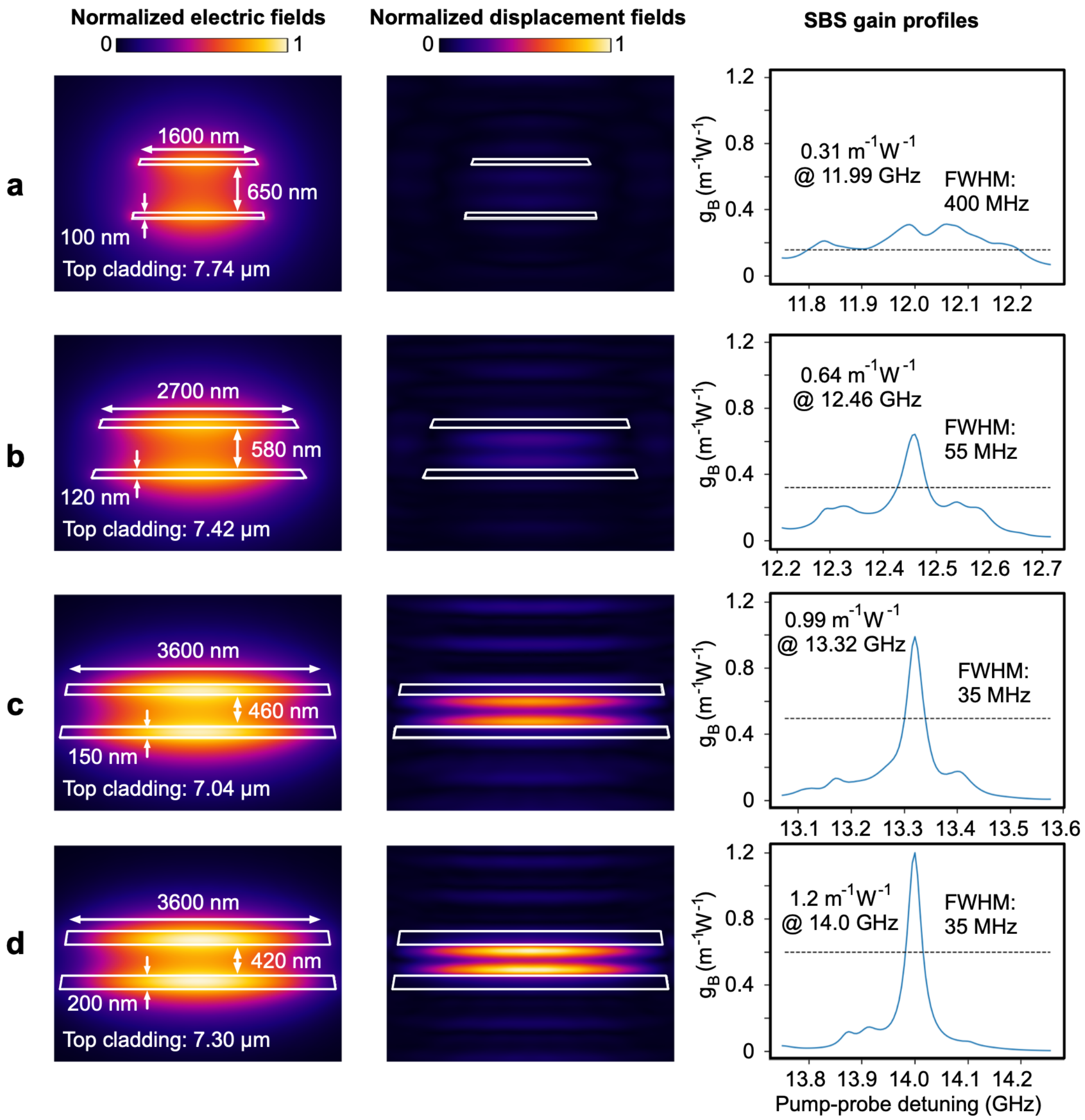

Fig. S5. **A selection of simulated geometries with SBS gain of 0.31, 0.64, 0.99, and 1.20 $m^{-1}W^{-1}$.** They are indicated in Fig. S4 by blue dots labeled **a**–**d**. Shown in this figure from left to right are simulated normalized electric field, displacement field, and SBS gain profiles. Parameters of each geometry are presented in the normalized electric field.

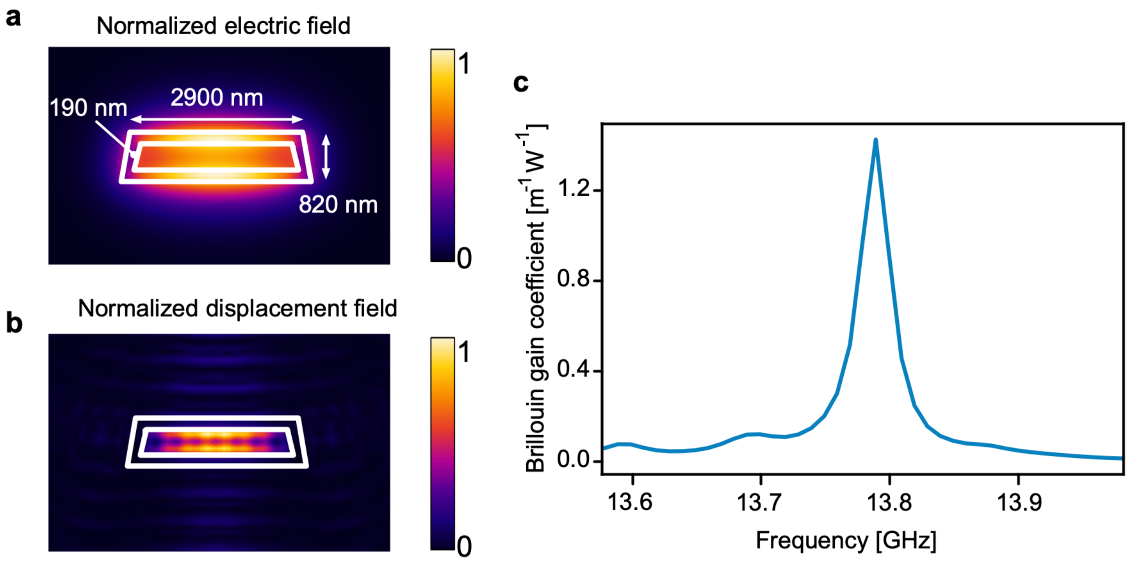

Fig. S6. **Simulation results of the box-shaped waveguide.** **a**. Normalized electric field, **b**. normalized displacement field, and **c** Brillouin gain spectrum.

Box-shaped Waveguide

We also explored a different variety of the multilayer waveguide for a higher Brillouin gain coefficient. One possible solution is to use the box-shaped waveguide, i.e., adding sidewalls to improve acoustic confinement. Fig. S6 shows the normalized electric and acoustic displacement fields, as well as the Brillouin gain profile of a box-shaped waveguide. The geometry parameters are based on the optimized symmetric double-stripe structure. The peak of the Brillouin gain coefficient is at 1.4 $m^{-1}W^{-1}$, which is slightly higher than that of a double stripe waveguide with the same geometry parameters. However, optical propagation losses in these waveguides are also higher, due to roughness in the sidewalls. Hence, in this work we focus on double-stripe waveguide, given the best combination of the Brillouin gain coefficient and the propagation loss obtained using the double-stripe structure.

Benchmarking and Comparison with Previous Results

To verify our simulation model, we also benchmark our model with the geometries shown in [15] and [48]. Fig. S7a and b show our simulated normalized electric field and normalized displacement field for the waveguide in [15], which match quite well with the corresponding fields provided in the supplementary material of [15]. The peak Brillouin gain coefficient and the trend also match results in [15]. The acoustic frequency at the peak shifts from the results in [15] by about 300 MHz, which may be due to the different material properties applied in models. Our simulated normalized electric field, normalized displacement field, and SBS gain profile of the waveguide in [48], as shown Fig. S7d and e, are quite close to original simulated and experimental results in [48] as well.

Table S2 compares the double-stripe waveguide with recently reported silicon nitride [15, 48], silicon [16], and chalcogenide [17] waveguides. Our waveguide has low propagation loss, reasonable Brillouin gain coefficient, negligible two-photon absorption, and non-suspended structure, making it a unique choice for higher density integration of Brillouin circuits in a standard process.

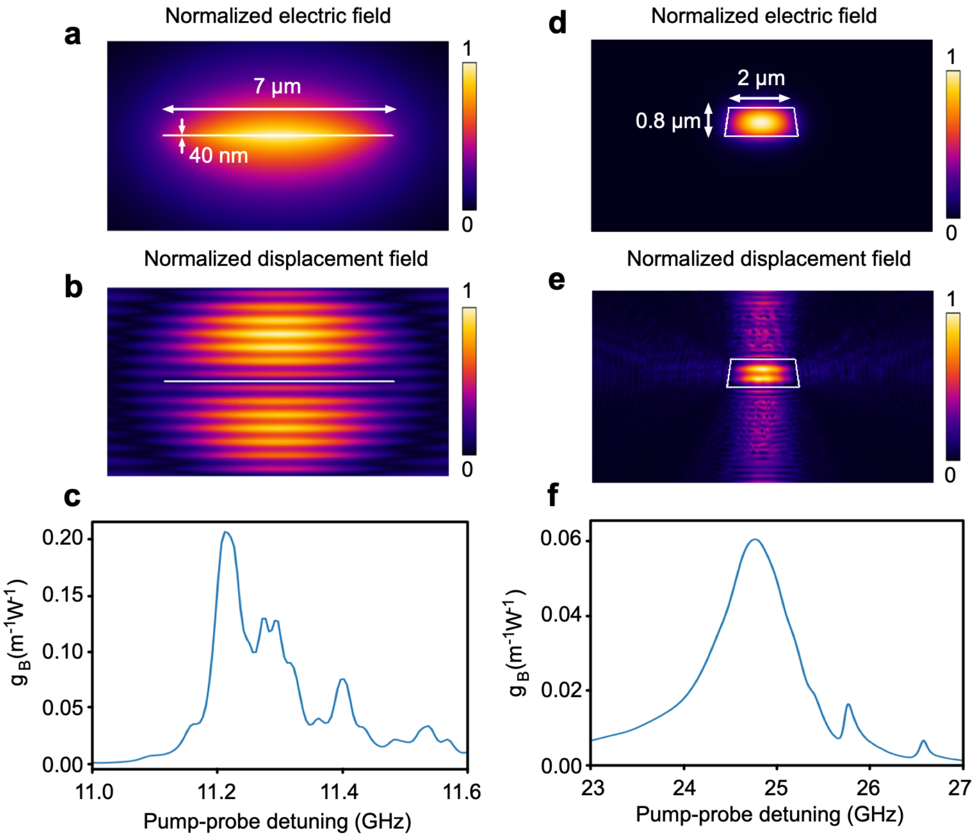

Fig. S7. **Benchmark with the geometries in [15] and [48].** **a**, Normalized electric field, **b**, normalized displacement field, and **c**, SBS gain profile of the waveguide in [15] simulated in our model. **d**, Normalized electric field, **e**, normalized displacement field, and **f**, SBS gain profile of the waveguide in [48] simulated in our model.

TABLE S2. **Characteristics of different integrated SBS platforms.**

**Platform**	**Propagation loss** (dB/m)	**Brillouin gain coefficient** ($m^{-1}W^{-1}$)	**SBS linewidth** (MHz)	**Brillouin shift frequency** (GHz)	**Type of SBS**	**Two-photon absorption**	**Acoustic guidance**	**Integration density**
Dilute Si_3N_4 [15]	0.4	0.1	143	10.9	Backward	No	No	+
Silicon [16]	26	400	13.1	6.02	Forward	Yes	Yes	+++
Chalcogenide [17]	20	303.4	34	7.7	Backward	No	Yes	+
Thick Si_3N_4 [48]	<10	0.07	517	25	Backward	No	Yes	+++
This work	19.2	0.40	130	12.93	Backward	No	Yes	+++

SUPPLEMENTARY NOTE B: DETAILS OF THE EXPERIMENTS

This supplementary note describes the details of the experiments, as well as the methods used to extract the Brillouin properties from the measurement results.

SBS Gain Characterisations

In the gain characterisation setup, the probe laser is a Toptica DFB pro BFY laser, which operates around 1550 nm. We scan the laser wavelength using current control. The electric coefficient of this laser is 0.8 GHz/mA. The laser output is modulated using a Thorlabs LN05S-FC intensity modulator, with a 10.075 MHz sine wave generated using a Hewlett-Packard 33120A function/arbitrary waveform generator (AWG). After modulation the light is amplified by an Amonics AEDFA-PA-35 amplifier.

The pump laser is an Avanex A1905LMI, also operating around 1550 nm. Its output is modulated using a Covega LN81S-FC intensity modulator, driven by a 10 MHz sine wave produced by a Wiltron 69147A Synthesized Sweep Generator. The light is then amplified using an Amonics AEDFA-33-B amplifier. To prevent crosstalk from signal mixing, the reference signal is created by mixing the synchronized output of the AWG (a TTL square wave at the same frequency as the sine wave) with the 10 MHz reference output of the sweep generator. These signals are mixed using a Mini-Circuits ZFM-3H mixer. The probe light is sent to a Discovery Semiconductor DSC30S photodiode, after which the signal is sent to an EG&G Princeton Applied Research model 5510 lock-in amplifier which measures the amplitude of the signal. The main experimental parameters for the SBS characterization setup is in Table S3.

Fig. S8 shows the same chip response as in Fig. 2b, but this time over a wider detuning range to include the response of the fiber pigtail. This fiber is a 1.4 m length single mode fiber (SMF), which has a Brillouin gain of 0.14 $m^{-1}W^{-1}$ [66]. The amplitude of the fiber response is used to calculate the SDS Brillouin gain coefficient, as described in the Methods section of the main text.

TABLE S3. The experimental parameters of the SBS characterization setup.

Parameter	Value	Unit	Description
P_{probe}	21.3	dBm	Probe optical power after amplification
P_{pump}	30.0	dBm	Pump optical power after amplification
$V_{\pi,\text{probe}}$	5.5	V	V_π of the probe modulator
$V_{\pi,\text{pump}}$	5.6	V	V_π of the pump modulator
$P_{\text{mod,probe}}$	16.0	dBm	RF power sent to probe modulator
$P_{\text{mod,pump}}$	6.0	dBm	RF power sent to pump modulator
r_{pd}	0.8	A/W	Photodiode sensitivity
α_{c}	2.35	dB/facet	Coupling loss per facet, including fiber components

Detailed Results of SBS Gain Calculations

Table S4 compares the simulated and the measured Brillouin properties of the SDS waveguides investigated in this work. We observe excellent agreement between simulations and experiments in the Brillouin frequency shift and linewidth. The maximum discrepancy in the Brillouin frequency shift is 270 MHz, which is only 2% of the SBS shift (13 GHz). The discrepancies in linewidths are below 50 MHz, except for the narrowest waveguide of 1.1 µm. We observed larger discrepancies in the Brillouin gain coefficients. The measured results are consistently 24–50% lower than what is predicted from simulations, which can be explained by the fabrication uncertainties in layer thickness and waveguide width (See Supplementary Note A). Similar trends and accuracy are also presented in previously reported SBS experiments including waveguides in chalcogenides [67], silicon [67], and silicon nitride [15, 48].

To determine the linewidth of our measured Brillouin responses we fitted our experimental results to a Lorentzian function. The Lorentzian function is written as:

$$\mathcal{L}(f, A, \mu, \sigma) = \frac{A}{\pi}\left(\frac{\sigma}{(f-\mu)^2+\sigma^2}\right) \tag{S3}$$

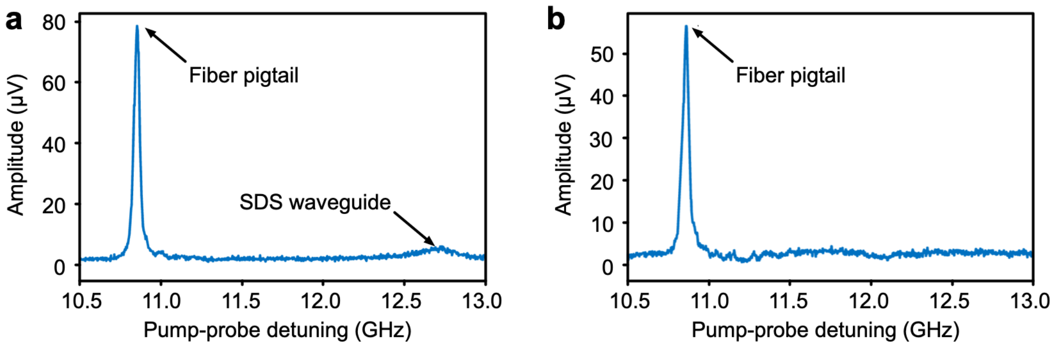

Fig. S8. **The measurements including the fiber response.** **a**, The measurement on the 50 cm long, 1.2 µm wide SDS waveguide. **b**, The measurement on the 50 cm long ADS waveguide.

TABLE S4. **Simulated and measured Brillouin properties of SDS waveguides.**

**Waveguide width** (µm)	**Propagation loss** (dB/cm)	**Effective length** (cm)	**Frequency shift** **Simulated** (GHz)	**Frequency shift** **Measured** (GHz)	**Linewidth** **Simulated** (MHz)	**Linewidth** **Measured** (MHz)	**Gain coefficient** **Simulated** ($m^{-1}W^{-1}$)	**Gain coefficient** **Measured** ($m^{-1}W^{-1}$)
1.1	0.228	17.67	12.95	12.68	380	490	0.34	0.20
1.2	0.223	17.98	12.99	12.74	330	290	0.38	0.24
1.4	0.206	19.11	13.03	12.76	260	250	0.46	0.25
1.5	0.230	17.55	13.05	12.81	230	180	0.50	0.25
3.0	0.195	19.91	13.18	12.93	130	130	0.53	0.40

where f is the frequency, A the amplitude, µ the center frequency and σ the half-width at half-maximum.

We used the *Python* package *lmfit* to perform the fitting. Because the measurements have a high noise floor, especially in the case of the lower gain waveguides, we added an offset to the Lorentzian model. The experimental results and the fitted curves can be seen in Fig. S9.

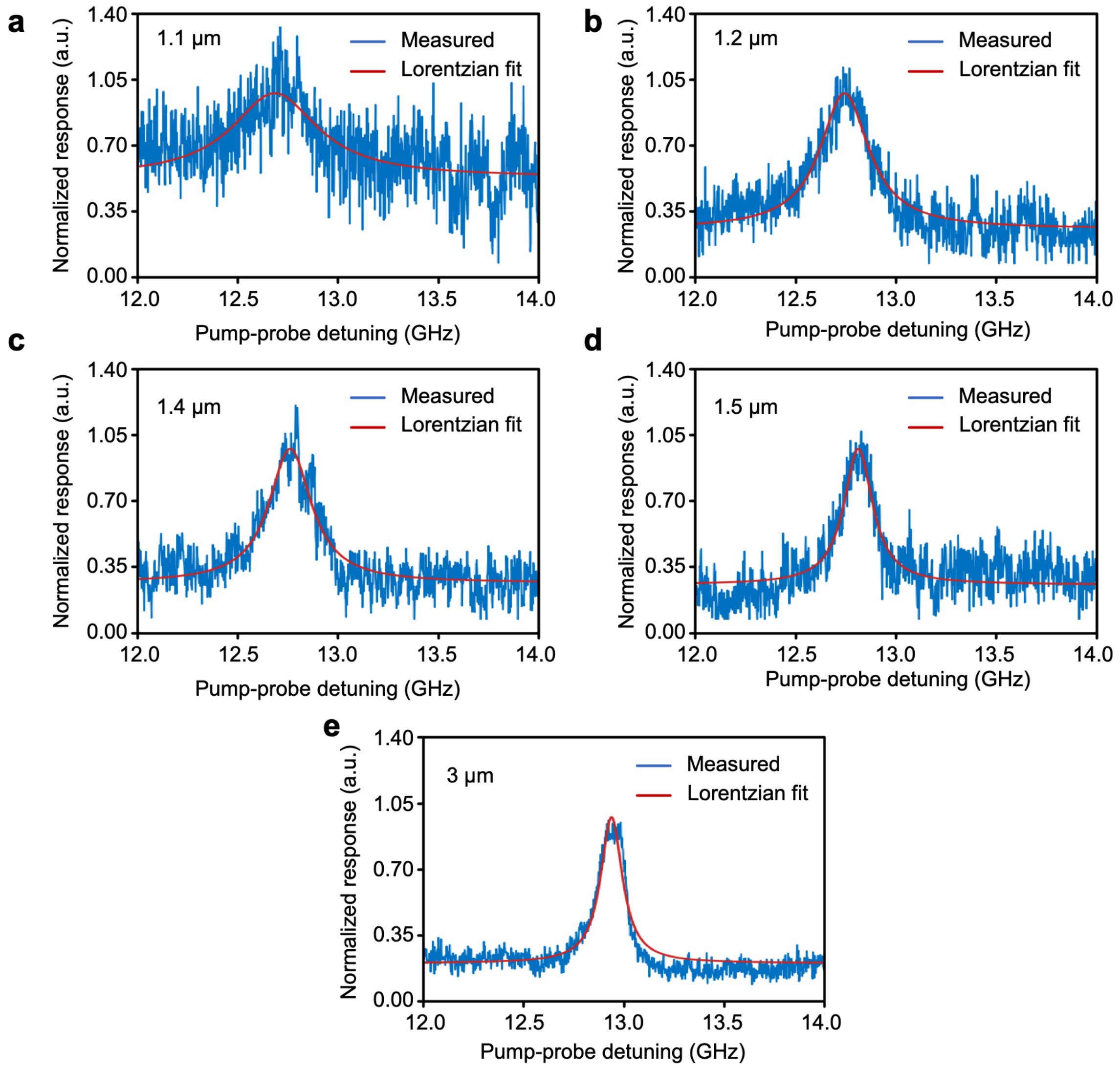

Fig. S9. **Fitting of the experimental results to extract the Brillouin linewidth values of various waveguide widths.** The amplitude axes are normalized to the height of the fitted line.

RF Photonic Notch Filter Experiments

In the RF photonic notch filter we used the same Toptica and Avanex lasers as probe and pump as described above. The RF response of the setup is measured using a Keysight P5007A (VNA).

The probe laser is modulated using a Covega LN81SFC modulator, and then sent to the first chip, which contains a bus with 8 all-pass ring resonators. These rings each have an FSR of 25 GHz, and are fully tunable. One of these rings is used in the signal processing, and the other 7 are disabled. After the ring resonators the light is amplified with an Amonics AEDFA-PA-35 amplifier, and sent to the second chip, which is SBS active. The medium is pumped by the Avanex laser, which is amplified using an amonics AEDFA-33-B amplifier. After the signal has passed through the gain chip it is filtered using an EXFO XTM-50-SCL-U tunable bandpass filter to remove unwanted pump reflections. After this the signal is amplified with a second Amonics AEDFA-PA-35 amplifier, and sent to a Discovery Semiconductor DSC30S photodiode. The main experimental parameters for the microwave photonic notch filter with a ring resonator are shown in Table S5.

We further measured the noise figure and dynamic range performances of the notch filter. A two-tone RF signal, centered at 2 GHz with a space of 10 MHz is generated from signal generators Wiltron 69147A and Rohde-Schwarz SMP02. This two-tone RF signal drives the intensity modulator with input power varies from 0.5 to 5 dBm, and the output of the notch filter is recorded with an RF spectrum analyzer (Keysight N9000B). As shown in Fig. S10, the measured spurious-free dynamic range (SFDR) of the notch filter is around 100.5 dB/Hz$^{2/3}$. The performances of this notch filter are summarized in Table S6.

TABLE S5. **The experimental parameters of the SBS notch filter setup with the ring resonator.**

Parameter	Value	Unit	Description
$P_{\rm RF}$	0	dBm	RF output power of VNA
$P_{\rm probe}$	21.3	dBm	Probe optical power after amplification
$P_{\rm pump}$	33.5	dBm	Pump optical power after amplification
$P_{\rm opt,pd}$	12.4	dBm	Optical power of secondary probe amplifier
V_π	5.6	V	V_π of the modulator
$r_{\rm pd}$	0.8	A/W	Photodiode sensitivity
$\alpha_{\rm c}$	2.35	dB/facet	Coupling loss per facet, including fiber components

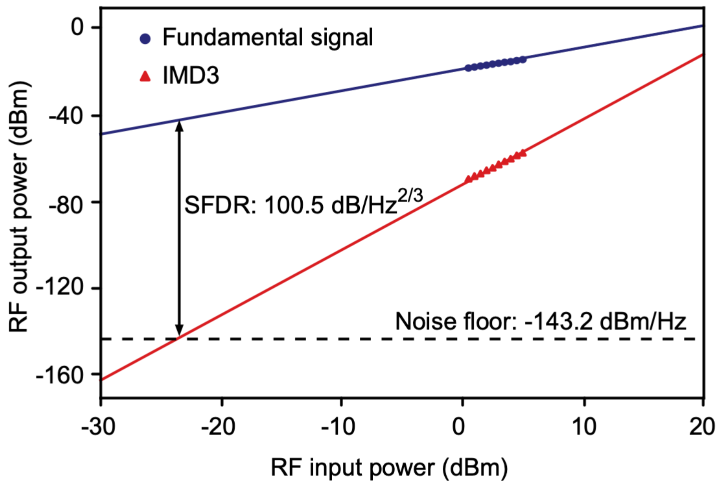

Fig. S10. Measured spurious-free dynamic range (SFDR) of the notch filter.

Alternative RF Photonic Notch Filter Using an IQ Modulator

The ring resonator RF photonic notch filter we discussed in the main text of this paper is one of two schemes we used to create an RF photonic notch filter. The second method also employs cancellation of the mixing products of

TABLE S6. Performance of the SBS notch filter with the ring resonator.

Link Gain dB	Noise Floor dBm/Hz	Noise Figure dB	Spur-Free Dynamic Range dB/Hz$^{2/3}$	Output Intercept Point dBm
-12.9	-143.2	43.7	100.5	7.6

TABLE S7. The experimental parameters of the SBS notch filter setup using an IQ modulator.

Parameter	Value	Unit	Description
P_{RF}	5.0	dBm	RF output power of VNA
P_{probe}	21.3	dBm	Probe optical power after amplification
P_{pump}	33.5	dBm	Pump optical power after amplification
$P_{\text{opt,pd}}$	13.5	dBm	Optical power of secondary probe amplifier
$V_{\pi,\text{IQ}}$	3.5	V	V_π of the IQ modulator
r_{pd}	0.8	A/W	Photodiode sensitivity
α_{c}	2.35	dB/facet	Coupling loss per facet, including fiber components

the sidebands to create a notch, but instead of a ring resonator it uses tailoring of the phase and amplitude of both optical sidebands [7, 52, 53]. A simplified schematic of this RF photonic notch filter is diagrammed in Fig. S11a. A key component in this filter is the in-phase quadrature (IQ) modulator (also known as the dual-parallel Mach-Zehnder modulator) used often synthesizing the RF modulates sidebands with the correct phase and amplitude relations prior to the narrowband processing using the SBS gain resonance. Table S7 lists the main experimental parameters of the SBS notch filter with the IQ modulator.

Fig. S11b shows the working principle of the RF photonics notch filter. The RF input (I) is modulated onto the probe laser using the IQ modulator creating an asymmetric dual sideband modulation, with the sidebands in anti-phase (II). The on-chip SBS interaction with the probe light then amplifies a spectral region of the lower sideband (III) making the sidebands equal in amplitude only at the frequency of the SBS peak gain. The processed signal was then sent to a photo-diode, resulting in an RF spectrum with a notch response due to the destructive interference between the mixing products of the sidebands and the optical carrier (IV).

In creating this notch filter we used the 1.4 µm waveguide, and a pump power of 33.5 dBm. This results in an SBS gain of 0.4 dB. We then tuned the IQ modulator to synthesize two sidebands with opposite phase, and an amplitude of the lower sideband that was 0.4 dB higher than that of the upper sideband. The resulting RF photonics notch filter response is depicted in Fig. S11d. The peak rejection of the filter was measured to be 30 dB and the 3 dB-bandwidth of the filter was 400 MHz.

This filter results in a notch that is less than half as wide as the notch created with the ring resonator setup, but the rejection rate is much lower. This is the result of using the asymmetric double sideband which leads to a much lower signal in the passband of the filter. The setup requires an additional optical amplifier to get to the -50 dB transmission that was observed in the ring resonator setup. Increasing the Brillouin gain by optimizing the waveguide's Brillouin gain coefficient and propagation losses requires a larger amplitude difference between the sidebands, resulting in a filter with a higher passband.

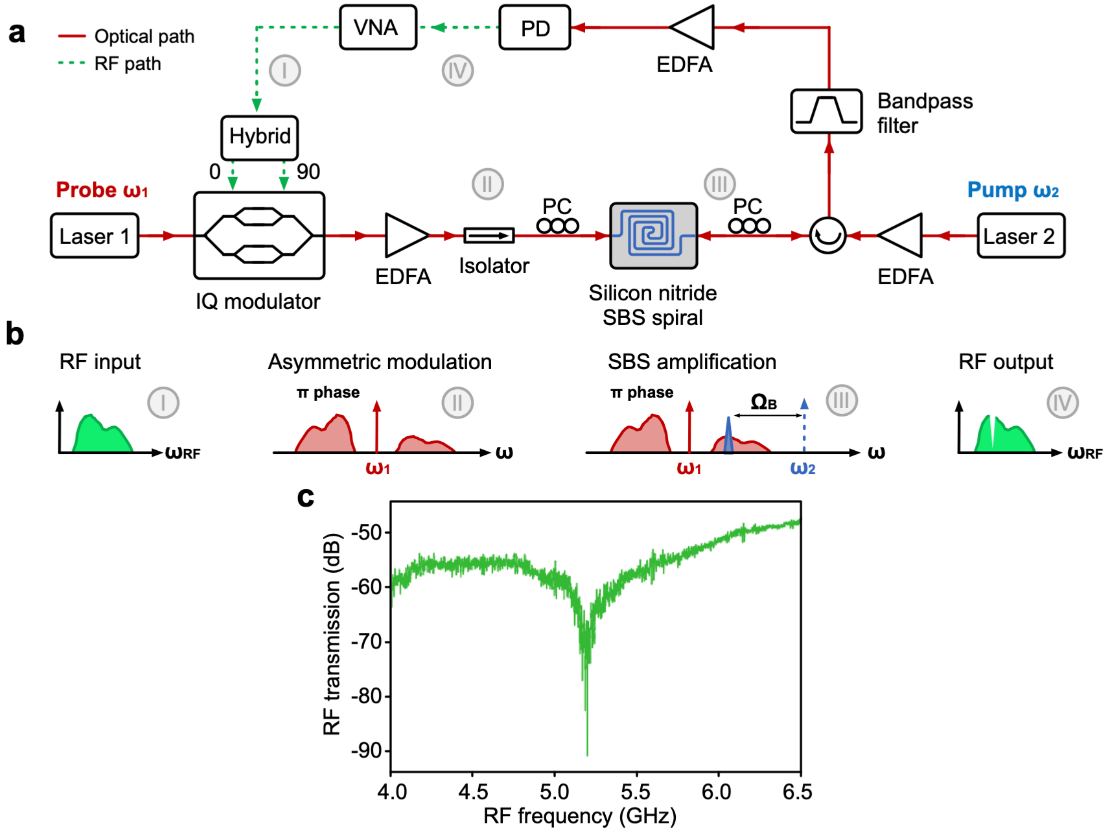

Fig. S11. **Microwave photonic notch filter using IQ modulator.** **a**, Schematic of the experimental setup to demonstrate the MWP notch filter. Hybrid: 90° RF hybrid coupler, PC: fiber polarization controller. **b**, RF and optical spectra at different points of signal path: (I) Input RF signal. (II) Asymmetric dual sideband phase modulated signal generated from the IQ modulator. The RF sidebands are out of phase and their amplitude ratio is controlled to match the SBS gain magnitude generated from the silicon nitride waveguide. (III) SBS gain from the silicon nitride waveguide is used equalize the sideband amplitue at the intended RF notch frequency. (IV) At the detector the mixing products between sidebands and the optical carrier leads to a notch filter due to RF cancellation. **c**, The measured high-rejection RF photonics notch filter response which was obtained using only 0.4 dB of on-chip SBS gain. The 3 dB bandwidth of the filter is 400 MHz and the rejection is 30 dB.

SUPPLEMENTARY NOTE C: ESTIMATION OF SBS LASER THRESHOLD

In this supplementary note we discuss the feasibility of a Brillouin laser based on the waveguide designs described in Supplementary Note A. To do so we describe the boundary condition for the resonator, the threshold condition and give the loss for different scenarios of propagation loss, from the measured samples and below.

An integrated Brillouin laser consists of a ring resonator pumped resonantly with an external pumping laser whilst on a resonance at the Brillouin downshifted frequency light can build up within the cavity. The restriction of both pump and the SBS shifted light needing to be resonant gives the condition: $\Omega/2\pi = n \cdot \mathrm{FSR}_\nu$ with n an integer and FSR_ν the free spectral range. We can describe the lasing threshold for the first mode [15] and write it as:

$$P_{\mathrm{th}} = \frac{\kappa^{-1}}{g_{\mathrm{B}} L} \left(\frac{\pi \Delta\nu_{\mathrm{L}}}{\mathrm{FSR}_\nu} \right)^2 \tag{S4}$$

where $\Delta\nu_L$ is the loaded linewidth of the resonator, L the length of the resonator and κ the coupling efficiency, not to be confused with decay rate or coupling strength. The coupling efficiency is given here as the ratio of the energy coupling rate ($2\pi\Delta\nu_{\mathrm{ext}}$) to the total decay rate ($2\pi\Delta\nu_{\mathrm{L}}$), i.e., $\kappa = \Delta\nu_{\mathrm{ext}}/\Delta\nu_{\mathrm{L}}$. By taking the loaded linewidth to be approximately the sum of the intrinsic linewidth and the coupling rate, i.e., $\Delta\nu_{\mathrm{L}} = \Delta\nu_{\mathrm{ext}} + \Delta\nu_0$, the coupling rate can be optimized for minimal lasing threshold leading to a coupling efficiency of $\kappa = 1/3$.

Finally the intrinsic linewidth can be converted to a propagation loss via the mean field approximation, i.e., $2\pi\Delta\nu_0 \approx v_{\mathrm{g}} \frac{\alpha}{10\log_{10}(e)}$ with v_{g} the group velocity and α the propagation loss in dB/m. Assuming optimized coupling for minimal threshold, the threshold power can now be expressed as:

$$P_{\mathrm{th}} = 0.089 \frac{\alpha^2 L}{g_{\mathrm{B}}}. \tag{S5}$$

Table S8 shows the calculated threshold power (eq.(S5)) for various values of the optical propagation loss ranging from 0.22 dB/cm to 0.01 dB/cm for the four different SDS waveguide configurations a–d selected in supplementary A and shown in Fig. S5. Also listed in table S8 are the group index calculated using the method given in [68] and the length L of the rings calculated using $\Omega/2\pi = \mathrm{FSR} = \frac{\mathrm{c}}{\mathrm{n_g L}}$.

TABLE S8. Laser threshold calculated for waveguide geometries a–d as shown in Fig. S5 for a Brillouin laser with a free spectral range matching the Brillouin shift.

**Geometry**	**Group index**	**FSR** (GHz)	**Length** (mm)	Peak g_{B} ($\mathrm{m^{-1}W^{-1}}$)	P_{th}($\alpha = 0.22$ dB/cm) (dBm)	P_{th}($\alpha = 0.15$ dB/cm) (dBm)	P_{th}($\alpha = 0.1$ dB/cm) (dBm)	P_{th}($\alpha = 0.05$ dB/cm) (dBm)	P_{th}($\alpha = 0.01$ dB/cm) (dBm)
a	1.603	11.99	15.60	0.31	33.4	30.1	26.5	20.5	6.5
b	1.656	12.46	14.53	0.64	29.9	26.6	23.1	17.1	3.1
c	1.723	13.22	13.16	0.99	27.6	24.3	20.8	14.7	0.8
d	1.804	14.00	11.87	1.20	26.3	23.0	19.5	13.4	-0.5

REFERENCES AND NOTES

1. M. Aspelmeyer, T. J. Kippenberg, F. Marquardt, Cavity optomechanics. *Rev. Mod. Phys.* **86**, 1391–1452 (2014).

2. A. H. Safavi-Naeini, D. van Thourhout, R. Baets, R. van Laer, Controlling phonons and photons at the wavelength scale: Integrated photonics meets integrated phononics. *Optica* **6**, 213 (2019).

3. B. J. Eggleton, C. G. Poulton, P. T. Rakich, M. J. Steel, G. Bahl, Brillouin integrated photonics. *Nat. Photonics* **13**, 664–677 (2019).

4. R. W. Boyd, *Nonlinear Optics* (Elsevier Science Publishing Co. Inc., 2008).

5. P. T. Rakich, C. Reinke, R. Camacho, P. Davids, Z. Wang, Giant enhancement of stimulated Brillouin scattering in the subwavelength limit. *Phys. Rev. X* **2**, 11008 (2012).

6. D. Marpaung, J. Yao, J. Capmany, Integrated microwave photonics. *Nat. Photonics* **13**, 80–90 (2019).

7. D. Marpaung, B. Morrison, M. Pagani, R. Pant, D.-Y. Choi, B. Luther-Davies, S. J. Madden, B. J. Eggleton, Low-power, chip-based stimulated Brillouin scattering microwave photonic filter with ultrahigh selectivity. *Optica* **2**, 76 (2015).

8. J. Li, H. Lee, K. J. Vahala, Microwave synthesizer using an on-chip Brillouin oscillator. *Nat. Commun.* **4**, 1–7 (2013).

9. S. Gertler, E. A. Kittlaus, N. T. Otterstrom, P. T. Rakich, Tunable microwave-photonic filtering with high out-of-band rejection in silicon. *APL Photonics* **5**, 096103 (2020).

10. N. T. Otterstrom, E. A. Kittlaus, S. Gertler, R. O. Behunin, A. L. Lentine, P. T. Rakich, Resonantly enhanced nonreciprocal silicon Brillouin amplifier. *Optica* **6**, 1117–1123 (2019).

11. J. Li, K. Vahala, M.-G. Suh, Microresonator Brillouin gyroscope. *Optica* **4**, 346–348 (2017).

12. Y. H. Lai, M. G. Suh, Y. K. Lu, B. Shen, Q. F. Yang, H. Wang, J. Li, S. H. Lee, K. Y. Yang, K. Vahala, Earth rotation measured by a chip-scale ring laser gyroscope. *Nat. Photonics* **14**, 345–349 (2020).

13. G. Bashan, H. H. Diamandi, Y. London, E. Preter, A. Zadok, Optomechanical time-domain reflectometry. *Nat. Commun.* **9**, 1–9 (2018).

14. A. Denisov, M. A. Soto, L. Thévenaz, Going beyond 1000000 resolved points in a Brillouin distributed fiber sensor: Theoretical analysis and experimental demonstration. *Light Sci. Appl.* **5**, e16074 (2016).

15. S. Gundavarapu, G. M. Brodnik, M. Puckett, T. Huffman, D. Bose, R. Behunin, J. Wu, T. Qiu, C. Pinho, N. Chauhan, J. Nohava, P. T. Rakich, K. D. Nelson, M. Salit, D. J. Blumenthal, Sub-hertz fundamental linewidth photonic integrated Brillouin laser. *Nat. Photonics* **13**, 60–67 (2018).

16. N. T. Otterstrom, R. O. Behunin, E. A. Kittlaus, Z. Wang, P. T. Rakich, A silicon Brillouin laser. *Science* **360**, 1113–1116 (2018).

17. I. V. Kabakova, R. Pant, D.-Y. Y. Choi, S. Debbarma, B. Luther-Davies, S. J. Madden, B. J. Eggleton, Narrow linewidth Brillouin laser based on chalcogenide photonic chip. *Opt. Lett.* **38**, 3208–3211 (2013).

18. H. Al-Taiy, J. Klinger, N. Wenzel, S. Preußler, T. Schneider, Ultra-narrow linewidth, stable and tunable laser source for optical communication systems and spectroscopy. *Opt. Lett.* **39**, 5826–5829 (2014).

19. E. A. Kittlaus, W. M. Jones, P. T. Rakich, N. T. Otterstrom, R. E. Muller, M. Rais-Zadeh, Electrically driven acousto-optics and broadband non-reciprocity in silicon photonics. *Nat. Photonics* **15**, 43–52 (2020).

20. E. A. Kittlaus, N. T. Otterstrom, P. Kharel, S. Gertler, P. T. Rakich, Non-reciprocal interband Brillouin modulation. *Nat. Photonics* **12**, 613–619 (2018).

21. D. B. Sohn, S. Kim, G. Bahl, Time-reversal symmetry breaking with acoustic pumping of nanophotonic circuits. *Nat. Photonics* **12**, 91–97 (2018).

22. J. Kim, M. C. Kuzyk, K. Han, H. Wang, G. Bahl, Non-reciprocal Brillouin scattering induced transparency. *Nat. Phys.* **11**, 275–280 (2015).

23. R. Pant, C. G. Poulton, D.-Y. Choi, H. Mcfarlane, S. Hile, E. Li, L. Thevenaz, B. Luther-Davies, S. J. Madden, B. J. Eggleton, On-chip stimulated Brillouin scattering. *Opt. Express* **19**, 8285–8290 (2011).

24. R. van Laer, B. Kuyken, D. van Thourhout, R. Baets, Interaction between light and highly confined hypersound in a silicon photonic nanowire. *Nat. Photonics* **9**, 199–203 (2015).

25. H. Shin, J. A. Cox, R. Jarecki, A. Starbuck, Z. Wang, P. T. Rakich, Control of coherent information via on-chip photonic-phononic emitter-receivers. *Nat. Commun.* **6**, 2–9 (2015).

26. E. A. Kittlaus, H. Shin, P. T. Rakich, Large Brillouin amplification in silicon. *Nat. Photonics* **10**, 463–467 (2016).

27. E. A. Kittlaus, N. T. Otterstrom, P. T. Rakich, On-chip inter-modal Brillouin scattering. *Nat. Commun.* **8**, 1–9 (2017).

28. M. Merklein, I. V. Kabakova, T. F. S. Büttner, D. Y. Choi, B. Luther-Davies, S. J. Madden, B. J. Eggleton, Enhancing and inhibiting stimulated Brillouin scattering in photonic integrated circuits. *Nat. Commun.* **6**, 6396 (2015).

29. S. Li, X. Li, W. Zhang, J. Chen, W. Zou, Investigation of Brillouin properties in high-loss doped silica waveguides by comparison experiment. *IEEE Photon. Technol. Lett.* **32**, 948–951 (2020).

30. L. Chang, A. Boes, P. Pintus, J. D. Peters, M. J. Kennedy, X. W. Guo, N. Volet, S. P. Yu, S. B. Papp, J. E. Bowers, Strong frequency conversion in heterogeneously integrated GaAs resonators. *APL Photonics* **4**, 036103 (2019).

31. Q. Liu, H. Li, M. Li, Electromechanical Brillouin scattering in integrated optomechanical waveguides. *Optica* **6**, 778–785 (2019).

32. M. W. Puckett, K. Liu, N. Chauhan, Q. Zhao, N. Jin, H. Cheng, J. Wu, R. O. Behunin, P. T. Rakich, K. D. Nelson, D. J. Blumenthal, 422 Million intrinsic quality factor planar integrated all-waveguide resonator with sub-MHz linewidth. *Nat. Commun.* **12**, 934 (2021).

33. D. T. Spencer, J. F. Bauters, J. E. Bowers, M. J. R. Heck, Integrated waveguide coupled Si_3N_4 resonators in the ultrahigh-Q regime. *Optica* **1**, 153–157 (2014).

34. A. Kordts, J. D. Jost, M. H. P. Pfeiffer, M. Geiselmann, M. Zervas, T. J. Kippenberg, V. Brasch, Photonic Damascene process for integrated high-Q microresonator based nonlinear photonics. *Optica* **3**, 20–25 (2016).

35. K. Wörhoff, R. G. Heideman, A. Leinse, M. Hoekman, TriPleX: A versatile dielectric photonic platform. *Adv. Opt. Technol.* **4**, 189–207 (2015).

36. L. Zhuang, D. Marpaung, M. Burla, W. Beeker, A. Leinse, C. Roeloffzen, Low-loss, high-index-contrast Si_3N_4/SiO_2 optical waveguides for optical delay lines in microwave photonics signal processing. *Opt. Express* **19**, 23162–23170 (2011).

37. C. G. H. Roeloffzen, P. W. L. van Dijk, L. Zhuang, R. G. Heideman, R. M. Oldenbeuving, C. Taballione, L. S. Wevers, M. Benelajla, M. Hoekman, Y. Fan, D. Marchenko, J. P. Epping, K.-J. Boller, R. Dekker, A. van Rees, K. Worhoff, R. B. Timens, D. Marpaung, Y. Liu, C. Taddei, E. J. Klein, R. Grootjans, D. Geuzebroek, A. Alippi, A. Leinse, I. Visscher, E. Schreuder, D. Geskus, Low-loss Si3N4 TriPleX optical waveguides: technology and applications overview. *IEEE J. Select. Topics Quant. Electron.* **24**, 4400321 (2018).

38. C. G. H. Roeloffzen, L. Zhuang, C. Taddei, A. Leinse, R. G. Heideman, P. W. L. van Dijk, R. M. Oldenbeuving, D. A. I. Marpaung, M. Burla, K.-J. Boller, Silicon nitride microwave photonic circuits. *Opt. Express* **21**, 22937–22961 (2013).

39. C. Xiang, J. E. Bowers, P. A. Morton, Ultra-narrow linewidth laser based on a semiconductor gain chip and extended Si_3N_4 Bragg grating. *Opt. Lett.* **44**, 3825–3828 (2019).

40. Y. Fan, A. van Rees, C. G. H. Roeloffzen, D. Geskus, J. Mak, K.-J. Boller, M. Hoekman, P. J. M. van der Slot, R. M. Oldenbeuving, Hybrid integrated InP-Si_3N_4 diode laser with a 40-Hz intrinsic linewidth. *Opt. Express* **28**, 21713–21728 (2020).

41. W. Jin, Q. F. Yang, L. Chang, B. Shen, H. Wang, M. A. Leal, L. Wu, M. Gao, A. Feshali, M. Paniccia, K. J. Vahala, J. E. Bowers, Hertz-linewidth semiconductor lasers using CMOS-ready ultra-high-Q microresonators. *Nat. Photonics* **15**, 346–353 (2021).

42. B. Shen, L. Chang, J. Liu, H. Wang, Q. F. Yang, C. Xiang, R. N. Wang, J. He, T. Liu, W. Xie, J. Guo, D. Kinghorn, L. Wu, Q. X. Ji, T. J. Kippenberg, K. Vahala, J. E. Bowers, Integrated turnkey soliton microcombs. *Nature* **582**, 365–369 (2020).

43. A. L. Gaeta, M. Lipson, T. J. Kippenberg, Photonic-chip-based frequency combs. *Nat. Photonics* **13**, 158–169 (2019).

44. B. Stern, X. Ji, Y. Okawachi, A. L. Gaeta, M. Lipson, Battery-operated integrated frequency comb generator. *Nature* **562**, 401–405 (2018).

45. D. J. Moss, R. Morandotti, A. L. Gaeta, M. Lipson, New CMOS-compatible platforms based on silicon nitride and Hydex for nonlinear optics. *Nat. Photonics.* **7**, 597–607(2013).

46. D. Marpaung, C. Roeloffzen, R. Heideman, A. Leinse, S. Sales, J. Capmany, Integrated microwave photonics. *Laser Photonics Rev.* **7**, 506–538 (2013).

47. Y. Liu, Z. Qiu, X. Ji, A. Lukashchuk, J. He, J. Riemensberger, M. Hafermann, R. N. Wang, J. Liu, C. Ronning, T. J. Kippenberg, A photonic integrated circuit–based erbium-doped amplifier. *Science* **376**, 1309–1313 (2022).

48. F. Gyger, J. Liu, F. Yang, J. He, A. S. Raja, R. N. Wang, S. A. Bhave, T. J. Kippenberg, L. Thévenaz, Observation of stimulated Brillouin scattering in silicon nitride integrated waveguides. *Phys. Rev. Lett.* **124**, 013902 (2020).

49. C. G. Poulton, R. Pant, B. J. Eggleton, Acoustic confinement and stimulated Brillouin scattering in integrated optical waveguides. *J. Opt. Soc. Am. B* **30**, 2657 (2013).

50. W. T. Grubbs, R. A. MacPhail, High resolution stimulated Brillouin gain spectrometer. *Rev. Sci. Instrum.* **65**, 34–41 (1994).

51. Y. Liu, A. Choudhary, D. Marpaung, B. J. Eggleton, Integrated microwave photonic filters. *Adv. Opt. Photonics* **12**, 485–555 (2020).

52. D. Marpaung, B. Morrison, R. Pant, B. J. Eggleton, Frequency agile microwave photonic notch filter with anomalously-high stopband rejection. *Opt. Lett.* **38**, 4300–4303 (2013).

53. D. Marpaung, B. Morrison, R. Pant, C. Roeloffzen, A. Leinse, M. Hoekman, R. Heideman, B. J. Eggleton, Si_3N_4 ring resonator-based microwave photonic notch filter with an ultrahigh peak rejection. *Opt. Express* **21**, 23286–23294 (2013).

54. Y. Liu, D. Marpaung, A. Choudhary, B. J. Eggleton, Lossless and high-resolution RF photonic notch filter. *Opt. Lett.* **41**, 5306–5309 (2016).

55. Y. Liu, A. Choudhary, G. Ren, K. Vu, B. Morrison, A. Casas-Bedoya, T. Nguyen, D.-Y. Choi, P. Ma, A. Mitchell, S. Madden, D. Marpaung, B. Eggleton, Integration of Brillouin and passive circuits for enhanced radio-frequency photonic filtering. *APL Photonics* **4**, 106103 (2019).

56. S. Gertler, N. T. Otterstrom, M. Gehl, A. L. Starbuck, C. M. Dallo, A. T. Pomerene, D. C. Trotter, A. L. Lentine, P. T. Rakich, Narrowband microwave-photonic notch filters using Brillouin-based signal transduction in silicon. *Nat. Commun.* **13**, 1947 (2022).

57. O. Daulay, R. Botter, D. Marpaung, On-chip programmable microwave photonic filter with an integrated optical carrier processor. *OSA Continuum.* **3**, 2166 (2020).

58. X. Guo, Y. Liu, T. Yin, B. Morrison, M. Pagani, O. Daulay, W. Bogaerts, B. J. Eggleton, A. Casas-Bedoya, D. Marpaung, Versatile silicon microwave photonic spectral shaper. *APL Photonics* **6**, 036106 (2021).

59. M. W. Harrington, G. M. Brodnik, T. C. Briles, J. R. Stone, R. H. Streater, S. B. Papp, D. J. Blumenthal, Kerr Soliton Microcomb Pumped by an Integrated SBS Laser for Ultra-Low Linewidth WDM Sources, in *Optical Fiber Communication Conference (OFC) 2020 (2020), paper T4G.6* (Optica

Publishing Group, 2020), vol. Part F174-OFC 2020, p. T4G.6; https://opg.optica.org/abstract.cfm?uri=OFC-2020-T4G.6.

60. G. Lin, Q. Song, Kerr frequency comb interaction with Raman, Brillouin, and second order nonlinear effects. *Laser Photonics Rev.* **16**, 2100184 (2022).

61. Y. Bai, M. Zhang, Q. Shi, S. Ding, Z. Xie, X. Jiang, M. Xiao, Brillouin-Kerr soliton frequency combs in an optical microresonator. *Phys. Rev. Lett.* **126**, 63901 (2021).

62. I. H. Do, D. Kim, D. Jeong, D. Suk, D. Kwon, J. Kim, J. H. Lee, H. Lee, Self-stabilized soliton generation in a microresonator through mode-pulled Brillouin lasing. *Opt. Lett.* **46**, 1772–1775 (2021).

63. G. P. Agrawal, *Nonlinear Fiber Optics* (Elsevier Science Publishing Co. Inc., ed. 5, 2013).

64. J. Håkansson, D. van Thourhout, Generating novel waveguides for stimulated Brillouin scattering with genetic algorithms. *APL Photonics* **4**, 10803 (2019).

65. A. Kobyakov, M. Sauer, D. Chowdhury, Stimulated Brillouin scattering in optical fibers. *Adv. Opt. Photonics* **2**, 1–59 (2010).

66. B. C. P. Sturmberg, K. B. Dossou, M. J. A. Smith, B. Morrison, C. G. Poulton, M. J. Steel, Finite element analysis of stimulated Brillouin scattering in integrated photonic waveguides. *J. Lightwave Technol.* **37**, 3791–3804 (2019).

67. S. Afshar V, T. M. Monro, A full vectorial model for pulse propagation in emerging waveguides with subwavelength structures part I: Kerr nonlinearity. *Opt. Express* **17**, 2298–2318 (2009).

68. M. J. A. Smith, B. T. Kuhlmey, C. M. de Sterke, C. Wolff, M. Lapine, C. G. Poulton, Metamaterial control of stimulated Brillouin scattering. *Opt. Lett.* **41**, 2338–2341 (2016).
